# Editorial: Mechanobiology of biofilms and associated host -pathogen interactions

**DOI:** 10.3389/fcimb.2024.1416131

**Published:** 2024-04-23

**Authors:** Tagbo H. R. Niepa, Landon W. Locke, Timothy E. Corcoran, Janet S. Lee

**Affiliations:** ^1^ Department of Chemical Engineering, Carnegie Mellon University, Pittsburgh, PA, United States; ^2^ Department of Biomedical Engineering, Carnegie Mellon University, Pittsburgh, PA, United States; ^3^ Department of Biomedical Engineering, The Ohio State University, Columbus, OH, United States; ^4^ Division of Pulmonary, Allergy, and Critical Care Medicine, University of Pittsburgh, Pittsburgh, PA, United States; ^5^ Division of Pulmonary and Critical Care Medicine, Washington University School of Medicine, St. Louis, MO, United States

**Keywords:** biofilm, cell aggregates, interfacial film, rheology, infection, mechanobiology

## Biofilms: impacts, properties, and research advances

Biofilms are the most ubiquitous form of microbial life. They are commonly known for their harmful impacts on human health, such as invading the lungs of individuals with Cystic Fibrosis, or for the economic burden they cause while fouling environmental, industrial, and medical surfaces. Often described as complex fluids owing to their viscoelastic properties, biofilms are investigated across various disciplines (Klapper et al., 2002; Wilking et al., 2011; Gordon et al., 2017). Researchers are exploring how physical forces influence the growth, development, physiology, and behavior of individual cells, as well as interactions within microbial communities and within hosts. In the context of human health, the mechanical attributes of these viscoelastic biomaterials are dynamic, evolving throughout the course of disease progression and confounding efforts aimed at infection control (Hall et al., 2014; Stewart, 2014). As research in this field continues to expand, it becomes crucial to examine the recent advances made in the fight against biofilm-associated infections.

## Featured articles in this Research Topic

The articles in this Research Topic shed light on the mechanobiology of biofilms, offering perspectives and insights that enrich our comprehension of the viscoelastic properties of biofilm infections, mechanical interactions with immune cells, interfacial rheology of mixed species, and the role of biofilm-derived molecules in mediating interspecies and interkingdom microbial interactions. For instance, the article by Wells et al. delves into the intriguing concept of biofilm mechanobiology, offering a fresh perspective on how biofilms formed by both gram-positive and gram-negative microorganisms function. These microbial communities are encased in a self-produced matrix of polymers, proteins, and other biomolecules, forming a protective shield against external threats such as antibiotics and immune clearance. This, in turn, contributes to persistent infections. The unique viscoelastic properties of biofilms pose significant challenges to the immune system, necessitating a deeper understanding of biofilm mechanics to unravel their role in disease persistence. The article underscores the importance of investigating biofilm mechanics to develop novel therapeutic strategies, using *Pseudomonas aeruginosa* as a compelling example to encourage further exploration in this promising field.

The mechanobiology of biofilms has emerged as a multidisciplinary field at the interface of biology, engineering, chemistry, and physics. While traditional methods like bulk rheology (Gloag et al., 2020) and microrheology (Rogers et al., 2008) remain prevalent, novel techniques are emerging to explore biofilm interfacial properties, including those at fluid interfaces (Balmuri et al., 2020; Prasad et al., 2023). The study by Balmuri et al. employed innovative approaches to manipulate biofilm mechanics. Utilizing pendant drop elastometry and imaging, researchers characterized the mechanical properties and structural integrity of bacterial films at hexadecane-water interfaces. These methods facilitated the evaluation of mucolytic agents in disrupting the biofilm matrix and modifying film elasticity. Additionally, the study investigated the effects of these agents on the viscoelastic properties of biofilms in scenarios involving both cooperation and competition between *P. aeruginosa* and *S. aureus*.

Rhamnolipids, biosurfactants synthesized by *P. aeruginosa*, mediate interactions among diverse bacterial species, particularly in the “great divide” that segregates *P. aeruginosa* and *S. aureus* in mixed biofilms. Unraveling the mechanisms driving this segregation holds promise for innovative strategies to modulate biofilm composition and improve therapeutic outcomes. In the study led by Bru et al., intriguing insights into the interplay between these bacterial strains were unveiled. Firstly, *P. aeruginosa* swarms were observed to be repelled by colonies of clinical *S. aureus* isolates, resulting physical separation. This phenomenon was attributed to *S. aureus*-produced phenol-soluble modulins (PSMs) that form amyloid fibrils. However, rhamnolipids produced by *P. aeruginosa* allow both bacteria to coexist by creating distinct microenvironments. This interaction, along with the influence of other molecules like *Bacillus subtilis* surfactant, highlights the complex dynamics of bacterial coexistence.

Fungal pathogens are an emerging threat, particularly in immunocompromised individuals. Similar to bacterial infections, infections attributed to *Candida* spp. are linked to implanted medical devices and prostheses, where *Candida* cells have the propensity to form resilient biofilms. Recognizing the need for new antifungal treatments due to drug resistance, Powell et al. investigated the potential synergistic antibiofilm properties of the natural product alginate oligosaccharide OligoG combined with nystatin against 13 *Candida* strains. *In vitro* testing showed that this combination reduced the biovolume of established biofilms and induced greater cell death. Although the precise anti-biofilm mechanism of OligoG is unclear, it is hypothesized to chelate calcium, disrupting biofilm structures, as demonstrated in *P. aeruginosa* (Powell et al., 2018). Interestingly, OligoG may possess mechanical-altering properties against *Candida* biofilms, diminishing their defenses against immune cells. The convergence of combination therapy, innovative antifungals, and the resultant mechanical alterations in fungal biofilms are expected to be pivotal areas of investigation in the forthcoming years.

Recent investigations challenge the conventional notion of *in vivo* biofilms as surface-attached communities, showing the formation of “suspended aggregates” in polymer-rich environments through physical forces. Known as “depletion aggregation,” this process occurs when bacteria encounter each other, restricting polymer movement and causing an osmotic imbalance, leading to physical cell adhesion (Marenduzzo et al., 2006). These aggregates have been observed in cystic fibrosis patients and wounds. Secor et al. showed exopolysaccharides sustain these aggregates post-depletion, with only *P. aeruginosa* strains overexpressing Pel or Psl retaining aggregative state via bridging-mediated aggregation. Depletion aggregation’s ability to foster antimicrobial-tolerant phenotypes raises concerns regarding persistent infections. Understanding its role in biofilm mechanobiology is crucial for elucidating microbial adhesion and aggregate formation mechanisms, impacting biofilm mechanics. Thus, further exploration of this mode is warranted for infection control strategies.

## Conclusion and perspectives

In summary, this Research Topic aims to inspire investment and growth in the relatively untapped field of biofilm mechanobiology, envisioning transformative approaches to combating infectious diseases and improve therapeutic efficacy by deciphering the mechanical cues governing biofilm dynamics. As we delve into the intricate phenomena governing biofilm formation, spanning from individual cells to cell aggregates and interfacial or surface-attached films, collaboration across disciplines and ongoing exploration of cutting-edge technologies will prove instrumental in unlocking the full potential of this captivating frontier in microbiology.

## Author contributions

TN: Writing – original draft, Writing – review & editing. LL: Writing – original draft, Writing – review & editing. TC: Writing – original draft, Writing – review & editing. JL: Writing – original draft, Writing – review & editing.
